# Novel and Potent Small Molecules against Melanoma Harboring BRAF Class I/II/III Mutants for Overcoming Drug Resistance

**DOI:** 10.3390/ijms22073783

**Published:** 2021-04-06

**Authors:** Namkyoung Kim, Injae Shin, Jiwon Lee, Eunhye Jeon, Younghoon Kim, Seongshick Ryu, Eunhye Ju, Wonjeong Cho, Taebo Sim

**Affiliations:** 1 KU-KIST Graduate School of Converging Science and Technology, Korea University, 145 Anam-ro, Seongbuk-gu, Seoul 02841, Korea; nkkim312@yuhs.ac (N.K.); sij5266@yuhs.ac (I.S.); yhoon9408@yuhs.ac (Y.K.); ssryu129@yuhs.ac (S.R.); jooeh127@gmail.com (E.J.); 2 Severance Biomedical Science Institute, Graduate School of Medical Science, Brain Korea 21 Project, Yonsei University College of Medicine, 50 Yonsei-ro, Seodaemun-gu, Seoul 03722, Korea; wldnjs513@yuhs.ac (J.L.); jeh6525@yuhs.ac (E.J.); esther153cho@yuhs.ac (W.C.)

**Keywords:** melanoma, vemurafenib-resistant, BRAF class I/II/III mutants, pan-class BRAF inhibitor, type-II kinase inhibitor

## Abstract

Melanoma accounts for the majority of skin cancer deaths. About 50% of all melanomas are associated with BRAF mutations. BRAF mutations are classified into three classes with regard to dependency on RAF dimerization and RAS signaling. The most frequently occurring class I BRAF V600 mutations are sensitive to vemurafenib whereas class II and class III mutants, non-V600 BRAF mutants are resistant to vemurafenib. Herein we report six pyrimido[4,5-*d*]pyrimidin-2-one derivatives possessing highly potent anti-proliferative activities on melanoma cells harboring BRAF class I/II/III mutants. Novel and most potent derivative, SIJ1777, possesses not only two-digit nanomolar potency but also 2 to 14-fold enhanced anti-proliferative activities compared with reference compound, GNF-7 against melanoma cells (SK-MEL-2, SK-MEL-28, A375, WM3670, WM3629). Moreover, SIJ1777 substantially inhibits the activation of MEK, ERK, and AKT and remarkably induces apoptosis and significantly blocks migration, invasion, and anchorage-independent growth of melanoma cells harboring BRAF class I/II/II mutations while both vemurafenib and PLX8394 have little to no effects on melanoma cells expressing BRAF class II/III mutations. Taken together, our six GNF-7 derivatives exhibit highly potent activities against melanoma cells harboring class I/II/III BRAF mutations compared with vemurafenib as well as PLX8394.

## 1. Introduction

Melanoma is a highly aggressive type of skin cancer with its worldwide incidence increasing over the recent 50 years [1,2,3]. Overall death rate of skin cancer is less than 5%, but melanoma is the major cause of skin cancer death [4,5]. Especially, the late stage of melanoma has a poor prognosis, with 25% of the recent 5-year relative survival rate in metastatic melanoma patients [6]. Targeted- and immuno- therapies have enhanced the patient survival [7]. Especially, BRAF and MEK inhibition to block the mitogen-activated protein kinase (MAPK) pathway by vemurafenib and dabrafenib provided the improved tumor response rate and progression-free survival. It is well known that the MAPK signaling pathway is activated in up to 80–90% of melanoma patients, through 20–80% of BRAF mutation or 25% of NRAS mutation [8].

Recent studies have categorized various BRAF mutations into three classifications in accordance with their dependency on RAS signaling activity and RAF dimerization. Class I BRAF mutation, represented by BRAF V600 mutants, is basically a RAS-independent monomer with high kinase activities. Class II BRAF mutations, such as G464 and G469 mutants, signal as RAS-independent constitutive dimers possessing intermediate to high kinase activity. On the other hand, class III BRAF mutations, including D594 and G466, are RAS-dependent heterodimers having impaired BRAF kinase activity.

Three BRAF V600 mutant inhibitors, namely, vemurafenib, dabrafenib and encorafenib, have been approved in combination with MEK inhibitors as a treatment regimen for patients with advanced melanoma harboring BRAF V600 mutants. Vemurafenib, one of the approved RAF inhibitors, selectively inhibits RAF monomers (class I, BRAF V600 mutants), while it confers resistance to class II and class III BRAF mutants. The efficacy of vemurafenib is limited due to the acquired resistance mechanism through which MAPK/ERK signaling pathway is paradoxically activated. To overcome drug resistance of vemurafenib, PLX8394 has been developed as a next generation RAF inhibitor that does not induce paradoxical ERK activation. However, this paradox breaker possesses limited effects against class II and class III BRAF mutants [9]. It should be noted that BRAF class I/II/III mutants are present in 65.9%, 11.4%, and 9.5% of melanoma patients, respectively [10]. At the moment, there are no approved targeted therapies for advanced melanoma patients harboring class II and class III non-V600 BRAF mutants. Consequently, there are great unmet needs for developing novel agents capable of overriding drug resistant melanoma harboring pan-BRAF mutants.

We have previously reported that GNF-7, a type-II multi-targeted kinase inhibitor, has the capability to strongly inhibit Bcr-Abl T315I gatekeeper mutant [11] and also overcome NRAS mutant-driven acute myeloid lymphoma (AML) through suppressing ACK and GCK [12,13]. Generally, a type-II kinase inhibitor occupies the ATP-binding pocket of kinases in their inactive conformation (the DFG-out state) [14], whereas vemurafenib, a type-I inhibitor, occupies the ATP-binding pocket of BRAF in its active conformation (the DFG-in state). In our previous study, we reported [15] GNF-7 and its derivative SIJ1227 capable of strongly inhibiting melanoma cells with class I (A375)/class II (C8161) BRAF mutations and lung cancer cells with class II (H1755)/class III (H1666) BRAF mutations. Although vemurafenib has been approved as a treatment regimen for melanoma, it shows resistance to both class II and class III BRAF mutants. On the basis of our analysis, we, therefore, anticipate that it is worth carrying out further exploration of novel pan-class BRAF inhibitors and performing further research focused on melanoma in order to override vemurafenib-resistance. To this end, we herein describe the identification of novel kinase inhibitors possessing potent inhibitory activities against melanoma cells harboring class II (G464E) and class III (G469E, D594G) as well as class I (V600E) BRAF mutations.

## 2. Results and Discussion

### 2.1. Molecular Docking Study of SIJ1777 with BRAF V600E Mutant

**Figure 1:**
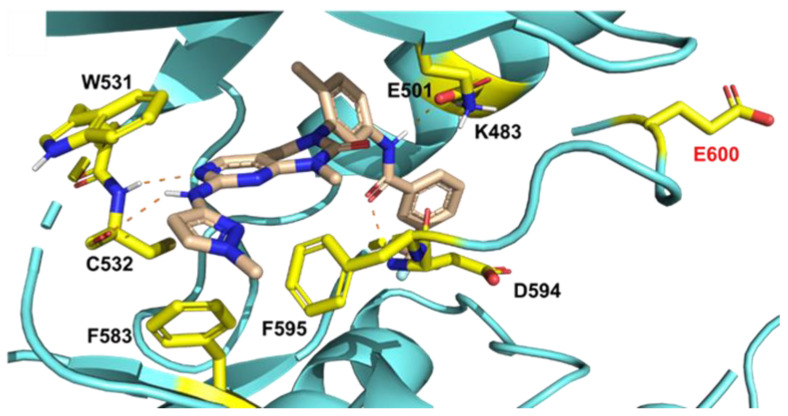
Docking model prediction of SIJ1777 on BRAF V600E mutant. H-bond interactions are indicated with dashed lines.

To investigate whether SIJ1777 would be active on BRAF V600E mutant, we carried out molecular docking studies using X-ray co-crystal structure (PDB: 4G9R) on BRAF V600E mutant (Figure 1). Analysis of the docking study revealed that SIJ1777 forms H-bonds with a backbone of C532 in the hinge region and also makes a pair of H-bonds with E501/D594 residues. Moreover, the pyrimidine ring of SIJ1777 participates in π–π stacking with W531 and F595, which might significantly contribute to the binding affinity. The molecular docking study suggests that SIJ1777 would be active on BRAF V600E mutant, which is consistent with the previous results of molecular docking studies of GNF-7 [15].

### 2.2. Six Derivatives Strongly Suppress Proliferation of Melanoma Cells Harboring Class I/II/III BRAF Mutations

**Figure 2:**
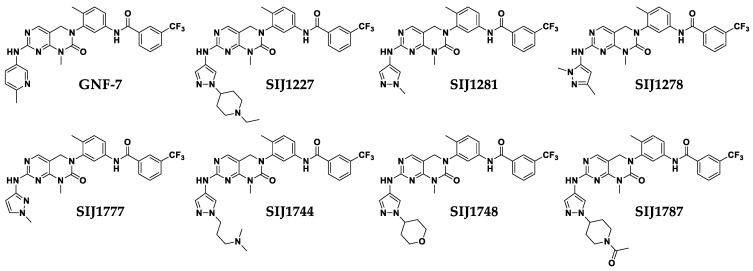
Chemical structures of GNF-7 and its derivatives.

Encouraged by our previous results [15], we designed and synthesized six GNF-7 derivatives possessing substituted pyrazole moieties as head group (Figure 2). We evaluated anti-proliferative activities of GNF-7, SIJ1227, and six derivatives on six melanoma cell lines. As the data shown in Table 1, all six derivatives possess excellent GI_50_ values on SK-MEL-28 (GI_50_s = 0.02 to 0.15 μM), A375 (GI_50_s = 0.02 to 0.09 μM), C8161 (GI_50_s = 0.02 to 0.13 μM), WM3670 (GI_50_s = 0.03 to 0.15 μM), and WM3629 (GI_50_s = 0.03 to 0.12 μM). Furthermore, all derivatives strongly suppress proliferation of SK-MEL-2 (NRAS Q61R activating activation, BRAF wt dimer) melanoma cells (GI_50_s = 0.02 to 0.24 μM), while vemurafenib and PLX8394 have almost no anti-proliferative activities on SK-MEL-2 cells. (GI_50_s = 3.84 to 19.30 μM). It is notable that vemurafenib and PLX8394 displayed little to no effect on WM3670 (class III BRAF G469E, GI_50_s = 13.65 to 17.86 μM) and WM3629 (class III BRAF D594G, GI_50_s = 27.95 to 33.10 μM) as well as on C8161 (class II BRAF G464E, GI_50_s = 5.81 to 27.30 μM), even though vemurafenib has strong inhibitory activity on SK-MEL-28 and A375 cell lines (class I BRAF V600E) [16]. PLX8394, a novel “paradox breaker”, blocks ERK signaling by disrupting BRAF-containing dimer [9], but it has almost no anti-proliferative effect on SK-MEL-2, WM3670, and WM3629. Among six derivatives, SIJ1777 possesses not only two-digit nanomolar potencies in all melanoma cells tested but also more enhanced anti-proliferative activities than those of GNF-7 on SK-MEL-2 (~12 fold enhanced), SK-MEL-28 (~4 fold enhanced), A375 (~2 fold enhanced), WM3670 (~3 fold enhanced), and WM3629 (~5 fold enhanced). In addition, the in vitro cytotoxicity of SIJ1777 against normal skin fibroblast cell HFF-1 was lower (5 to 10 fold) than that on melanoma cells. Moreover, it is worth noting that our six derivatives are also capable of strongly suppressing other cancer cells harboring class II/III BRAF mutations such as H1755 non-small cell lung cancer (NSCLC) cells (class II BRAF G469A), MDA-MB-231 triple negative breast cancer (TNBC) cells (class II BRAF G464V), and H1666 NSCLC cells (class III BRAF G466V) (Table S1).

### 2.3. Effects of SIJ1777 on MAPK/AKT Signaling against Melanoma Cells Harboring BRAF wt or Class I/II/III Mutations

**Figure 3:**
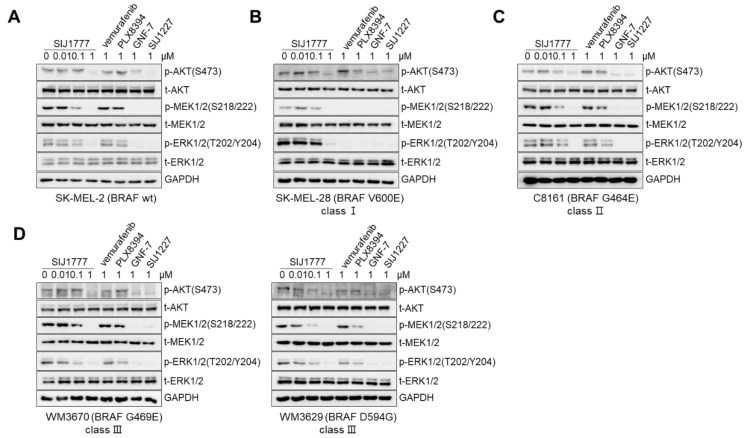
The effect of SIJ1777 on AKT and MAPK signaling pathways in melanoma cell lines harboring various BRAF mutation status (**A**) SK-MEL-2 (wt) (**B**) SK-MEL-28 (class I) (**C**) C8161 (class II) (**D**) WM3670, WM3629 (class III). Cells were treated with 0.01, 0.1, 1 μM of SIJ1777, and 1 μM of vemurafenib, PLX8394, GNF-7, and SIJ1227 for 2 h. Cell lysates were subjected to western blot analysis to estimate the phospho- or total- form of AKT, MEK, ERK levels, and GAPDH was used as the internal loading controls.

It has been reported that increased phosphorylation of AKT is correlated with BRAF inhibitor resistance based on data obtained from melanoma patients tissue samples [17]. Moreover, there have been several reports showing combined inhibition of both BRAF and AKT signaling might be beneficial in achieving anti-melanoma effects [15,18]. Thus, we evaluated the influence of SIJ1777 on MAPK and AKT signaling pathways in melanoma cell lines having different BRAF mutation statuses (wt or class I/II/III mutants). As shown in Figure 3, SIJ1777 completely suppressed phospho-MEK, -ERK, and -AKT levels at 1 μM concentration, regardless of BRAF mutation status in melanoma cells. In SK-MEL-2 (BRAF wt), C8161 (class II BRAF G464E), WM3670 (class III BRAF G469E), and WM3629 (class III BRAF D594G), 1 μM concentration of vemurafenib and PLX8394 could not inhibit the activities of MEK, ERK, and/or AKT, while SIJ1777 attenuated phosphorylation of MEK, ERK, and AKT completely at the same concentration. In SK-MEL-28 (class II BRAF V600E), vemurafenib and PLX8394 completely abolished p-MEK, p-ERK, but not p-AKT. In WM3629 (class III BRAF D594G), AKT and ERK inhibitory activities of SIJ1777 are higher than those of vemurafenib and PLX8394 and activation of both AKT and MAPKs were totally inhibited by 1 μM of SIJ1777 (Figure S1).

Consistent with our previous findings [15], these results provide additional evidence that blockade of both MAPK/AKT signaling could offer enhanced anti-proliferative activities of SIJ1777 on vemurafenib- and PLX8394- resistant melanoma cells.

### 2.4. Effects of SIJ1777 on Apoptosis Induction in Melanoma Cell Lines

**Figure 4:**
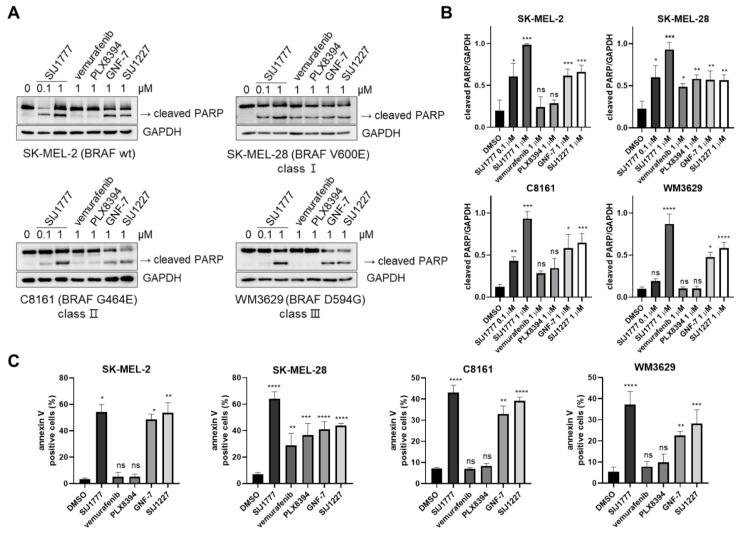
The effect of SIJ1777 on apoptosis induction. (**A**) Western blot for pro-apoptotic marker level (cleaved PARP) in melanoma cell lines. GAPDH was used as the internal loading control. (**B**) Quantification graphs of western blot results by ImageJ (*n* = 3). (**C**) Apoptotic cell (annexin V-positive) population was measured by flow cytometry analysis against melanoma cell lines (*n* = 3). Cells were treated with indicated substances for 24 h. Statistical significances were determined using a one-way ANOVA analysis (* *p* < 0.05, ** *p* < 0.01, *** *p* < 0.001, **** *p* < 0.0001).

In order to figure out whether the anti-proliferative effects of SIJ1777 are mainly due to apoptosis induction, we conducted a western blot assay to investigate the cleaved PARP level, one of the pro-apoptotic markers (Figure 4A,B). SIJ1777 increased cleaved PARP level in a concentration-dependent manner on melanoma cells (SK-MEL-2, SK-MEL-28, C8161, WM3629). However, vemurafenib and PLX8394 could not induce PARP cleavage in melanoma cells harboring BRAF wt (SK-MEL-2), class II (C8161), and class III (WM3629) mutants, which is in accordance with the fact that vemurafenib and PLX8394 have low anti-proliferative activities on those cells. We also conducted flow cytometry analysis after treating 1 μM of compounds to determine apoptotic cell population using annexin V/propidium iodide (PI) staining (Figure 4C, Figure S2). It was observed that SIJ1777 highly induces apoptosis against SK-MEL-2, C8161, and WM3629 cells. Vemurafenib and PLX8394 showed no significant induction of apoptosis in these melanoma cells. It is worthwhile to note that treatment of SIJ1777 induced an increase in apoptotic cells up to ~37% in WM3679 cells, while vemurafenib and PLX8394 displayed little effect on apoptosis induction. In the SK-MEL-28 cell line, SIJ1777 led to a strong increase in apoptotic cells up to ~64%, and the treatment of vemurafenib and PLX8394 also induced apoptosis up to ~30% and ~37%, respectively. Taken together, SIJ1777 exerts anti-proliferative effects via induction of apoptosis in melanoma cells harboring class I/II/II BRAF mutations.

### 2.5. Effects of SIJ1777 on Cellular Migration and Invasion Abilities in Melanoma Cell Lines

**Figure 5:**
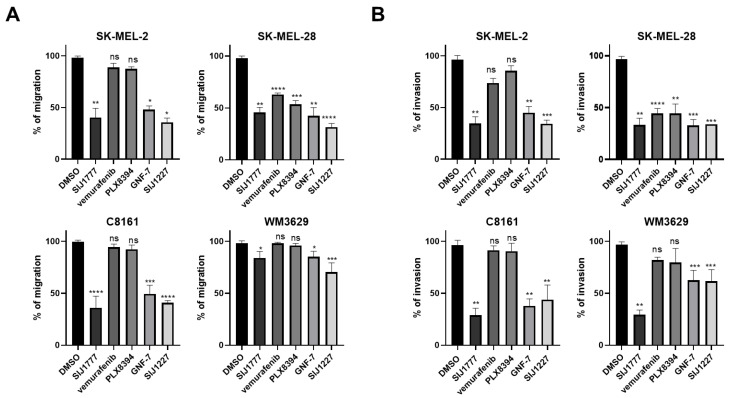
The effect of SIJ1777 on migration and invasion of melanoma cells harboring BRAF wt or class I/II/II mutations. (**A**) Scratch assay results for assessing migration capability. After scratching each cell monolayer, indicated compounds at 0.01 μM concentration were incubated for 12 h. Migration ratio was analyzed using migrated area using ImageJ (*n* = 3). (**B**) Boyden chamber assay for assessing invasion capability using cell invasion kit (QCM ECMatrix Cell Invasion Assay, *n* = 3). Statistical significances were determined using a one-way ANOVA analysis (* *p* < 0.05, ** *p* < 0.01, *** *p* < 0.001, **** *p* < 0.0001).

Previous studies have revealed that BRAF is associated with cellular migration and invasion activities in various types of cancer, including colon cancer [19], NSCLC [20], thyroid cancer [21], and melanoma [22]. Therefore, we assessed migration and invasion inhibitory activities of SIJ1777 in melanoma cells. As shown in Figure 5, migration and invasion capabilities of each cell are significantly downregulated by SIJ1777 at 0.01 μM concentration. Vemurafenib and PLX8394 decreased migration and invasion of SK-MEL-28 cells, while they showed little suppressive effect on SK-MEL-2, C8161, and WM3629 (Figures S3 and S4).

### 2.6. Colony Formation Inhibitory Activities of SIJ1777

**Figure 6:**
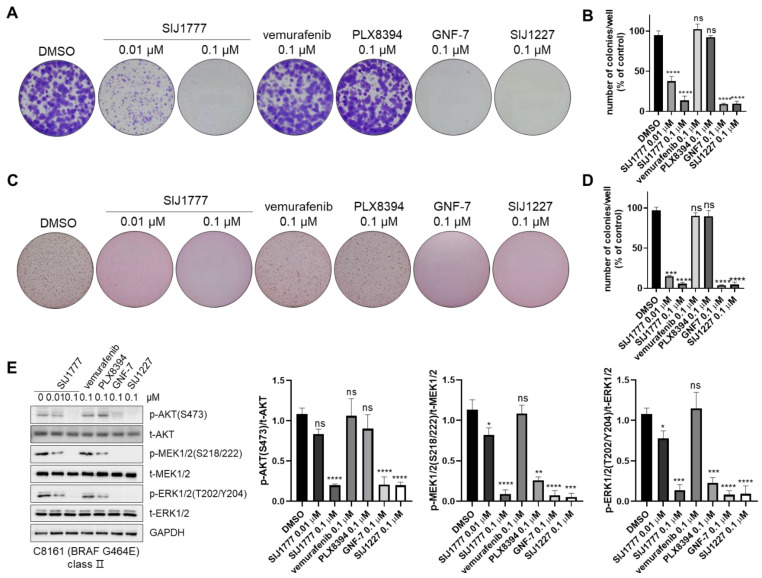
Clonogenic assay analysis of SIJ1777 in C8161. (**A**,**B**) 2D clonogenic assay (colony formation assay) results of the compounds on C8161 melanoma cell. After incubation with test compounds for 14 days, colonies were photographed without magnification. (**C**,**D**) 3D clonogenic assay (soft agar assay) results of test compounds on C8161 melanoma cell. Cells embedded within 0.35% low melting agar and incubated with the indicated compounds for 14 days and observed without magnification. (**B**,**D**) Number of colonies were determined automatically by ImageJ (*n* = 3, respectively). (**E**) Western blot analysis of SIJ1777 in C8161. Cells were treated with 0.01, 0.1 μM of SIJ1777, and 0.1 μM of vemurafenib, PLX8394, GNF-7, and SIJ1227 for 24 h. Cell lysates were subjected to western blot analysis to estimate the phospho- or total- form of AKT, MEK, ERK levels, and GAPDH was used as the internal loading controls (left panel). Quantification result (*n* = 3) of western blot result by ImageJ (right panel). Statistical significances were determined using a one-way ANOVA analysis (* *p* < 0.05, ** *p* < 0.01, *** *p* < 0.001, **** *p* < 0.0001).

Finally, we performed 2D and 3D clonogenic assays in C8161 to determine whether SIJ1777 could suppress tumorigenesis in melanoma cells (Figure 6A–D). Cells were incubated for 14 days with the indicated concentration of the compounds. SIJ1777 is remarkably capable of suppressing colony formation and anchorage-independent growth even at 0.01 μM concentration, while both vemurafenib and PLX8394 have little effect on colony formation at 0.1 μM concentration under 2D and 3D conditions. After 24 h treatment of 0.1 μM of SIJ1777, activities of MEK, ERK, and AKT were strongly downregulated (Figure 6E). These results indicate that blockage of both MAPK and AKT signaling by SIJ1777 prevents tumorigenesis of C8161 melanoma cells.

## 3. Materials and Methods

### 3.1. Chemistry

#### 3.1.1. General Information

Unless otherwise described, all commercial reagents and solvents were purchased from commercial suppliers and used without further purification. All reactions were performed under N_2_ atmosphere in flame-dried glassware. Reactions were monitored by TLC with 0.25 mm E. Merck precoated silica gel plates (60 F254). Reaction progress was monitored by TLC analysis using a UV lamp, ninhydrin, or *p*-anisaldehyde stain for detection purposes. All solvents were purified by standard techniques. Purification of reaction products was carried out by silica gel column chromatography using Kieselgel 60 Art. 9385 (230–400 mesh). The purity of all compounds was over 95% and mass spectra and purity of all compounds was analyzed using Waters LCMS system (Waters 2998 Photodiode Array Detector, Waters 3100 Mass Detector, Waters SFO System Fluidics Organizer, Water 2545 Binary Gradient Module, Waters Reagent Manager, Waters 2767 Sample Manager) using SunFireTM C18 column (4.6 × 50 mm, 5 μm particle size): solvent gradient = 60% (or 95%) A at 0 min, 1% A at 5 min. Solvent A = 0.035% TFA in H_2_O; Solvent B = 0.035% TFA in MeOH; flow rate: 3.0 (or 2.5) mL/min. ^1^H and ^13^C NMR spectra were obtained using Bruker 400 MHz FT-NMR (400 MHz for ^1^H and 100 MHz for ^13^C) spectrometer and Bruker 300 MHz FT-NMR (300 MHz for ^1^H and 75.5 MHz for ^13^C). Standard abbreviations are used for denoting the signal multiplicities.

##### *N*-(3-(7-((1-(1-Ethylpiperidin-4-yl)-1H-pyrazol-4-yl)amino)-1-methyl-2-oxo-1,4-dihydropyrimido[4,5-d]pyrimidin-3(2H)-yl)-4-methylphenyl)-3-(trifluoromethyl)benzamide (SIJ1227)

The synthesis of SIJ1227 was described in our previous report [15].

##### *N*-(3-(7-((1,3-Dimethyl-1H-pyrazol-5-yl)amino)-1-methyl-2-oxo-1,4-dihydropyrimido[4,5-d]pyrimidin-3(2H)-yl)-4-methylphenyl)-3-(trifluoromethyl)benzamide (SIJ1278)

The synthesis of SIJ1278 was described in our previous report [12].

##### *N*-(4-Methyl-3-(1-methyl-7-((1-methyl-1H-pyrazol-4-yl)amino)-2-oxo-1,4-dihydropyrimido[4,5-d]pyrimidin-3(2H)-yl)phenyl)-3-(trifluoromethyl)benzamide (SIJ1281)

The synthesis of SIJ1281 was described in our previous report [12].

##### *N*-(3-(7-((1-(3-(Dimethylamino)propyl)-1H-pyrazol-4-yl)amino)-1-methyl-2-oxo-1,4-dihydropyrimido[4,5-d]pyrimidin-3(2H)-yl)-4-methylphenyl)-3-(trifluoromethyl)benzamide (SIJ1744)

To a solution of *N*-(3-(7-chloro-1-methyl-2-oxo-1,4-dihydropyrimido[4,5-*d*]pyrimidin-3(2*H*)-yl)-4-methylphenyl)-3-(trifluoromethyl)benzamide [12] (100 mg, 0.211 mmol) in 2-butanol (2 mL) was added 1-(3-(dimethylamino)propyl)-1*H*-pyrazol-4-amine (39 mg, 0.232 mmol), K_2_CO_3_ (690 mg, 1.055 mmol), Xphos (20 mg, 0.042 mmol) and Pd_2_(dba)_3_ (40 mg, 0.042 mmol) at room temperature. The reaction mixture was then stirred for 1 h at 100 °C, cooled to room temperature, filtered and concentrated. The resulting residue was purified by silica gel column chromatography on silica gel (0% to 10% MeOH/DCM) to afford SIJ1744 (79 mg, 62%) as a pale gray solid. ^1^H NMR (400 MHz, DMSO-*d*_6_) δ 10.54 (s, 1H), 9.44 (bs, 1H), 8.30 (s, 1H), 8.26 (d, *J* = 8.07 Hz, 1H), 8.09 (s, 1H), 7.98 (d, *J* = 7.58 Hz, 1H), 7.87 (bs, 1H), 7.82–7.76 (m, 2H), 7.64 (dd, *J* = 8.31 Hz, 1.96 Hz, 1H), 7.53 (s, 1H), 7.31 (d, *J* = 8.31 Hz, 1H), 4.67 (d, *J* = 13.94 Hz, 1H), 4.49 (d, *J* = 13.94 Hz, 1H), 4.08 (t, *J* = 6.72 Hz, 2H), 2.19–2.15 (m, 2H), 2.13 (s, 3H), 2.12 (s, 6H), 1.91–1.81 (m, 2H); ^13^C NMR (75.5 MHz, DMSO-*d*_6_) δ 164.3, 159.1, 157.6, 153.9, 152.8, 141.7, 138.0, 136.1, 132.3, 131.4, 131.2, 130.3, 130.2, 129.9, 129.4, 128.7, 128.6, 126.3, 124.7, 124.7, 124.6, 124.6, 123.4, 122.6, 120.2, 120.1, 120.0, 119.8, 56.4, 49.9, 47.1, 45.6, 28.7, 28.4, 17.3. LRMS (ESI) *m*/*z* 608 [M + H]^+^.

##### *N*-(4-Methyl-3-(1-methyl-2-oxo-7-((1-(tetrahydro-2H-pyran-4-yl)-1H-pyrazol-4-yl)amino)-1,4-dihydropyrimido[4,5-d]pyrimidin-3(2H)-yl)phenyl)-3-(trifluoromethyl)benzamide (SIJ1748)

To a solution of *N*-(3-(7-chloro-1-methyl-2-oxo-1,4-dihydropyrimido[4,5-*d*]pyrimidin-3(2*H*)-yl)-4-methylphenyl)-3-(trifluoromethyl)benzamide [12] (100 mg, 0.211 mmol) in 2-butanol (2 mL) was added 1-(tetrahydro-2*H*-pyran-4-yl)-1*H*-pyrazol-4-amine (39 mg, 0.232 mmol), K_2_CO_3_ (690 mg, 1.055 mmol), Xphos (20 mg, 0.042 mmol) and Pd_2_(dba)_3_ (39 mg, 0.042 mmol) at room temperature. The reaction mixture was then stirred for 1 h at 100 °C, cooled to room temperature, filtered and concentrated. The resulting residue was purified by silica gel column chromatography on silica gel (0% to 10% MeOH/DCM) to afford SIJ1748 (100 mg, 62%) as a pale gray solid. ^1^H NMR (400 MHz, DMSO-*d*_6_) δ10.54 (s, 1H), 9.46 (bs, 1H), 8.31 (s, 1H), 8.26 (d, *J* = 7.82 Hz, 1H), 8.10 (s, 1H), 7.98 (d, *J* = 7.82 Hz, 1H), 7.93 (s, 1H), 7.84–7.74 (m, 2H), 7.64 (dd, *J* = 8.31 Hz, 1.96 Hz, 1H), 7.56 (s, 1H), 7.31 (d, *J* = 8.56 Hz, 1H), 4.68 (d, *J* = 13.94 Hz, 1H), 4.49 (d, *J* = 13.94 Hz, 1H), 4.41-4.31 (m, 1H), 3.99–3.91 (m, 2H), 3.45 (m, 2H), 2.13 (s, 3H), 1.99–1.88 (m, 4H); ^13^C NMR (100 MHz, DMSO-*d*_6_) δ 163.9, 158.7, 157.2, 152.3, 141.3, 137.6, 135.6, 131.9, 130.9, 130.8, 129.8, 129.7, 129.4, 129.1, 128.7, 128.2, 128.2, 128.1, 125.4, 124.2, 124.2, 123.0, 122.7, 119.8, 119.3, 117.5, 117.5, 117.5, 117.4, 66.0, 57.1, 46.7, 33.0, 30.7, 28.4, 16.8. LRMS (ESI) *m*/*z* 607 [M + H]^+^.

##### *N*-(4-Methyl-3-(1-methyl-7-((1-methyl-1H-pyrazol-3-yl)amino)-2-oxo-1,4-dihydropyrimido[4,5-d]pyrimidin-3(2H)-yl)phenyl)-3-(trifluoromethyl)benzamide (SIJ1777)

To a solution of *N*-(3-(7-chloro-1-methyl-2-oxo-1,4-dihydropyrimido[4,5-*d*]pyrimidin-3(2*H*)-yl)-4-methylphenyl)-3-(trifluoromethyl)benzamide [12] (100 mg, 0.211 mmol) in 2-butanol (2 mL) was added 1-methyl-1*H*-pyrazol-3-amine (23 mg, 0.232 mmol), K_2_CO_3_ (690 mg, 1.055 mmol), Xphos (20 mg, 0.042 mmol) and Pd_2_(dba)_3_ (40 mg, 0.042 mmol) at room temperature. The reaction mixture was then stirred for 1 h at 100 °C, cooled to room temperature, filtered and concentrated. The resulting residue was purified by silica gel column chromatography on silica gel (0% to 10% MeOH/DCM) to afford SIJ1777 (84 mg, 74%) as a pale gray solid. ^1^H NMR (300 MHz, DMSO-*d*_6_) δ 10.52 (s, 1H), 9.67 (s, 1H), 8.30 (s, 1H), 8.27 (d, *J* = 7.89 Hz, 1H), 8.10 (s, 1H), 7.97 (d, *J* = 7.70 Hz, 1H), 7.83–7.76 (m, 2H), 7.68–7.61 (m, 1H), 7.55 (d, *J* = 2.02 Hz, 1H), 7.31 (d, *J* = 8.44 Hz, 1H), 6.59 (d, *J* = 2.11 Hz, 1H), 4.68 (d, *J* = 13.94 Hz, 1H), 4.50 (d, *J* = 13.94 Hz, 1H), 3.74 (s, 3H), 3.33 (s, 3H), 2.13 (s, 3H); ^13^C NMR (100 MHz, DMSO-*d*_6_) δ 164.3, 159.1, 157.5, 153.8, 152.7, 148.5, 141.7, 138.0, 136.1, 132.3, 131.4, 131.2, 131.2, 130.3, 130.1, 129.8, 129.5, 129.2, 128.7, 128.6, 125.8, 124.6, 124.6, 123.1, 120.2, 119.8, 96.9, 47.1, 38.7, 28.7, 17.3. LRMS (ESI) *m*/*z* 537 [M + H]^+^.

##### *N*-(3-(7-((1-(1-Acetylpiperidin-4-yl)-1H-pyrazol-4-yl)amino)-1-methyl-2-oxo-1,4-dihydropyrimido[4,5-d]pyrimidin-3(2H)-yl)-4-methylphenyl)-3-(trifluoromethyl)benzamide (SIJ1787)

To a solution of *N*-(3-(7-Chloro-1-methyl-2-oxo-1,4-dihydropyrimido[4,5-*d*]pyrimidin-3(2*H*)-yl)-4-methylphenyl)-3-(trifluoromethyl)benzamide [12] (100 mg, 0.211 mmol) in 2-butanol (2 mL) was added 1-(4-(4-amino-1*H*-pyrazol-1-yl)piperidin-1-yl)ethan-1-one (48 mg, 0.232 mmol), K_2_CO_3_ (690 mg, 1.055 mmol), Xphos (20 mg, 0.042 mmol) and Pd_2_(dba)_3_ (48 mg, 0.042 mmol) at room temperature. The reaction mixture was then stirred for 1 h at 100 °C, cooled to room temperature, filtered and concentrated. The resulting residue was purified by silica gel column chromatography on silica gel (0% to 10% MeOH/DCM) to afford SIJ1787 (83 mg, 61%) as a pale gray solid. ^1^H NMR (300 MHz, DMSO-*d*_6_) δ 10.52 (s, 1H), 9.43 (bs, 1H), 8.31 (s, 1H), 8.27 (d, *J* = 7.89 Hz, 1H), 8.09 (s, 1H), 8.00–7.89 (m, 2H), 7.83-7.75 (m, 2H), 7.68–7.62 (m, 1H), 7.57 (s, 1H), 7.31 (d, *J* = 8.44 Hz, 1H), 4.73–4.62 (m, 1H), 4.54–4.31 (m, 3H), 3.90 (d, *J* = 13.48 Hz, 1H), 3.24–3.13 (m, 1H), 2.70 (t, *J* = 11.92 Hz, 1H), 2.13 (s, 3H), 2.09–1.95 (m, 5H), 1.94–1.79 (m, 1H), 1.70 (qd, *J* = 12.01, *J* = 4.13 Hz, 1H); ^13^C NMR (75.5 MHz, DMSO-*d*_6_) δ 168.2, 163.9, 158.7, 157.1, 153.4, 153.4, 152.3, 141.3, 137.6, 135.6, 131.8, 130.9, 130.7, 129.8, 129.8, 129.4, 129.0, 128.2, 128.2, 128.2, 128.1, 125.8, 124.2, 124.2, 124.1, 124.1, 123.0, 122.2, 119.8, 119.3, 117.6, 57.9, 46.7, 44.6, 32.5, 31.8, 28.3, 21.3, 16.8. LRMS (ESI) *m*/*z* 648 [M + H]^+^.

#### 3.1.2. Molecular Docking Study

X-ray co-crystal structures of BRAF V600E mutant (PDB code: 4G9R) were retrieved from the Protein Data Bank. The retrieved protein–ligand structures were loaded into Maestro software (Schrödinger Release 2020-4). Protein Preparation Wizard was used for addition of all hydrogens, assignment of bond orders, deletion of all water molecules, and filling of missing residue and loops. Restrained energy minimization was applied using the OPLS3e force field. Docking study of SIJ1777 on BRAF V600E mutant kinase domain was carried out using GLIDE module. SIJ1777 was prepared using the LigPrep module. A docking grid defining BRAF V600E mutant kinase domain was generated mainly considering the binding pocket of the inhibitor on BRAF V600E mutant.

### 3.2. Biology

#### 3.2.1. Cell Culture

A375, C8161, and HFF-1 were obtained from ATCC (Manassas, VA, USA). H1666, H1755, MDA-MB-231, SK-MEL-2, and SK-MEL-28 was purchased from KCLB (Seoul, Korea). WM3629, WM3670 was purchased from Rockland (Limerick, PA, USA). C8161, H1666, H1755, MDA-MB-231, SK-MEL-2, and SK-MEL-28 were cultured at RPMI1640. A375, HFF-1, WM3629 and WM3670 was cultured at DMEM, supplemented with 10% (*v*/*v*) fetal bovine serum (FBS), 1% (*v*/*v*) penicillin/streptomycin (Welgene, Seoul, Korea). Cells were incubated at 37 °C in a humidified 5% CO_2_ incubator.

#### 3.2.2. Anti-Proliferation Assay

Cells were seeded in a 96-well plate with a density of 5.0 × 10^3^ cells per well. After cellular attachment, a 3-fold serially diluted compound in DMSO was treated to the cells. After 72 h incubation at 37 °C, the cell viability was observed with CellTiter Glo (G7572, Promega, Madison, WI, USA). Fitted dose−response curves and GI_50_ values were obtained by Graphpad prism 6.0 software. All experiments were conducted in duplicate with three independent assays.

#### 3.2.3. Western Blot

p-ERK1/2 (#5174), t-ERK1/2 (#9102), p-AKT(S473) (#9271), PARP (#9542), and GAPDH (#5174) primary anti-bodies were purchased from Cell signaling technology (Danvers, MA, USA). p-MEK1/2 antibody (sc-81503) was purchased from Santa Cruz (Dallas, TX, USA). t-AKT (A18120), t-MEK1/2 (A4868) antibodies were purchased from Abclonal. HRP-conjugated goat anti-rabbit secondary antibody (SA002-500), HRP-conjugated goat anti-mouse secondary antibody (SA001-500) were purchased from Gendepot (Katy, TX, USA). 1 × 10^6^ cells/well were seeded in a 6-well plate. After cell adhesion, each compound was treated for 2 h and followed brief washing by ice-cold PBS twice. Cells were lysed with a NP40 buffer (50 mM Tris-HCl pH 7.4, 1% NP40, 2 mM EDTA, 150 mM NaCl) containing protease inhibitor cocktail (#11873580001, Roche, Indianapolis, IN, USA) and phosphatase inhibitor cocktail (#04906837001, Roche, Indianapolis, IN, USA). Each sample was loaded with an equal amount of protein and separated by SDS-PAGE gel. After transfer to nitrocellulose membrane it was blocked with 5% skim milk (in TBS/T). The membrane was incubated at 4 °C overnight with uniformly diluted primary antibodies at 1:1000 (*v*/*v*) in TBS/T. After incubation with secondary antibodies (1:10,000, *v*/*v*) for 1 h at room temperature, ECL solution was treated and chemiluminescence signals were detected by ImageQuant™ LAS 4000 (GE Healthcare, Uppsala, Sweden). Western blot images were quantified with ImageJ (*n* = 3).

#### 3.2.4. Flow Cytometry Analysis

Cells (2 × 10^6^ cells per sample) were incubated with indicated compounds for 24 h. For harvesting, cells were trypsinized and briefly washed with ice-cold PBS twice. Samples were stained with Alexa Fluor 488 conjugated annexin V (A13201, Thermo Fisher, Waltham, MA, USA) and propidium iodide (#556463, BD Biosciences, Bedford, MA, USA). To eliminate the debris and prevent false-positive or -negative results, unstained cells were excepted by gating. Thereafter, apoptotic cells were analyzed by FACS Accuri™ C6 Plus (BD Biosciences, Bedford, MA, USA).

#### 3.2.5. Migration and Invasion Assay

For migration assay, a scratch assay was performed. Each melanoma cells (2.0 × 10^5^ cells per well) were seeded in 24-well plates. After 24 h, cells were scratched with a SPLScar^TM^ Scratcher (SPL Life Sciences, Pocheon, Korea) and the detached cells were removed by PBS washing twice. Cells were incubated in complete media with 0.01 μM concentrations of each compound for 12 h. The images were acquired at 0 h, and 12 h incubation with 100× magnification, and percent of migration were accessed using ImageJ (*n* = 3).

For invasion assay, Boyden chamber assay was conducted using QCM ECMatrix Cell Invasion Assay kit (ECM 550, Sigma-Aldrich, Munich, Germany) according to the manufacturer’s instruction. Briefly, cells were seeded in the transwell chamber insert (8 μm pore size) at a density of 5.0 × 10^5^ cells per well after serum starvation for 12 h. The cells were incubated with 0.01 μM concentration of each compound for 24 h at 37 °C. The non-invaded cells were eliminated and followed the invaded cells staining. Cells were observed with 100 × magnification. Stained cells were dissolved in 10% acetic acid. After transferring the 96-well plate, optical density at 560 nm was measured by EnVision^®^ 2105 microplate reader (PerkinElmer, Waltham, MA, USA) and quantified % of invaded cells (*n* = 3).

#### 3.2.6. Colony Formation Assay

For 2D clonogenic assay, colony formation assay was conducted. 1000 cells per well were seeded in a 6-well plate. The cells were treated with indicated concentrations of compounds for 14 days at 37 °C and 5% CO_2_. Colonies were stained by crystal violet solution for 20 min. The entire area of each well was observed without magnification, and the number of colonies per well was counted using ImageJ software (*n* = 3).

For 3D clonogenic assay, soft agar assay was conducted. On the 0.7% bottom agar, cells were plated in a 6-well plate (5000 cells per well) with 0.35% low melting agar (#50101, Lonza, Basel, Switzerland) containing the complete media. The cells were incubated with test compounds for 14 days at 37 °C and 5% CO_2_. Colonies were stained by iodonitrotetrazolium chloride (I8377, Sigma-Aldrich, Munich, Germany) for 24 h. The whole area of each well was observed without magnification, and the number of colonies per well was determined using ImageJ software (*n* = 3).

#### 3.2.7. Statistical Analysis

All numerical data are shown as mean ± standard deviation (SD). Statistical significances were evaluated using a one-way ANOVA in Graphpad (San Diego, CA, USA) prism 6.0 (* *p* < 0.05, ** *p* < 0.01, *** *p* < 0.001, and **** *p* < 0.0001).

## 4. Conclusions

Metastatic melanoma is highly linked with poor prognosis and is considered a very aggressive, lethal form of cancer. Acquired resistance to conventional melanoma therapies (either targeted- or immuno- therapy) caused by various bypass signaling activation mechanisms, leads to limited efficacy of currently available BRAF inhibitors, vemurafenib and PLX8394. Thus, there is an urgent unmet medical need to develop novel agents overcoming acquired BRAF inhibitor resistance in melanoma. In this study, we report that SIJ1777, a novel GNF-7 derivative, possesses potent anti-cancer effects on melanoma cells harboring BRAF class I/II/III mutations. SIJ1777 significantly suppresses the proliferation of melanoma cells in vitro, regardless of BRAF mutation status. Also, SIJ1777 substantially inhibits the activation of MEK, ERK, and AKT on melanoma cells harboring BRAF class I/II/III mutations. Moreover, SIJ1777 is capable of inducing apoptosis and blocking significantly migration, invasion, and anchorage-independent growth of melanoma cells harboring BRAF class I/II/II mutations. It is worthwhile noting that both vemurafenib and PLX8394 have little to no effect on proliferation, activation of AKT and ERK, induction of apoptosis, migration and invasion, and colony formation- anchorage-independent growth in melanoma cells harboring BRAF class II/III mutations such as C8161, WM3670, and WM3629 cells.

Taken together, SIJ1777 turned out to be highly effective on melanoma cells harboring BRAF class II/III mutations as well as the BRAF class I mutation. Obviously, SIJ1777 is clearly superior to vemurafenib and PLX8394 not only in terms of cellular potency but also inhibitory effects on MAPK/AKT signaling, migration/invasion, and colony formation on melanoma cells harboring BRAF class II/III mutations. This study provides additional evidence that suppression of PI3K/AKT signaling pathway in addition to MAPK cascade could be an effective strategy to override drug resistant melanoma. SIJ1777 and its derivatives may serve as novel and promising BRAF inhibitors targeting melanoma cells expressing pan-class BRAF mutations.

## Figures and Tables

**Table 1:** Anti-proliferative activities of the six GNF-7 analogs against melanoma cell lines harboring BRAF wt or class I/II/III mutants.

Entry	GI_50_ (μM) ^a^					
	-	Class I	Class I	Class II	Class III	Class III
	BRAF wt	BRAF V600E	BRAF V600E	BRAF G464E	BRAF G469E	BRAF D594G
	SK-MEL-2	SK-MEL-28	A375	C8161	WM3670	WM3629
vemurafenib	3.84 ± 0.04	0.49 ± 0.01	0.18 ± 0.02	5.81 ± 0.24	13.65 ± 0.89	33.10 ± 3.61
PLX8394	19.30 ± 2.02 **	0.53 ± 0.01	0.10 ± 0.04	27.30 ± 1.34 **	17.86 ± 0.31	27.95 ± 2.52
GNF-7	0.23 ± 0.08 ***	0.15 ± 0.02 *	0.06 ± 0.00	0.02 ± 0.00 ****	0.13 ± 0.01 ****	0.21 ± 0.00 ***
SIJ1227	0.05 ± 0.01 **	0.05 ± 0.01 **	0.04 ± 0.01	0.05 ± 0.01 ****	0.04 ± 0.00 ***	0.08 ± 0.00 **
SIJ1281	0.12 ± 0.01 ***	0.03 ± 0.01 ***	0.02 ± 0.00 *	0.02 ± 0.01 ****	0.03 ± 0.01 ***	0.03 ± 0.00 ***
SIJ1278	0.24 ± 0.03 ***	0.14 ± 0.02 *	0.09 ± 0.02	0.08 ± 0.01 ***	0.15 ± 0.00 ****	0.12 ± 0.00 ***
SIJ1777	0.02 ± 0.00 ***	0.04 ± 0.01 **	0.03 ± 0.00 *	0.03 ± 0.01 ****	0.04 ± 0.00 ****	0.04 ± 0.00 ***
SIJ1744	0.12 ± 0.01 ***	0.08 ± 0.01 **	0.02 ± 0.01 *	0.13 ± 0.06 **	0.13 ± 0.00 ****	0.08 ± 0.02 ***
SIJ1748	0.06 ± 0.00 **	0.02 ± 0.00 **	0.02 ± 0.01 *	0.03 ± 0.01 ***	0.12 ± 0.01 **	0.11 ± 0.02 ***
SIJ1787	0.03 ± 0.00 **	0.15 ± 0.00 *	0.03 ± 0.01 *	0.04 ± 0.02 ****	0.13 ± 0.01 ****	0.07 ± 0.01 ***

^a^ GI_50_ represents the concentration which inhibits 50% of half-maximal growth. Each cell lines were treated with indicated compounds for 72 h. Averages with standard deviation (*n* = 3, duplicate) are presented. Statistical significances were determined using a one-way ANOVA analysis (* *p* < 0.05, ** *p* < 0.01, *** *p* < 0.001, **** *p* < 0.0001).

## Supplementary Materials

Supplementary materials can be found at https://www.mdpi.com/article/10.3390/ijms22073783/s1, Table S1: Anti-proliferative activities on cancer cells harboring class II/II BRAF mutations and skin fibroblast cells, Figure S1: Quantitative analysis of western blot, Figure S2: FACS analysis after 24 h treatment of SIJ1777 in melanoma cells, Figure S3: Migration inhibitory activity of SIJ1777 against melanoma cells, Figure S4: Invasion inhibitory activity of SIJ1777 against melanoma cells.

## Abbreviations

AML	Acute myeloid leukemia
MAPK	Mitogen-activated protein kinase
NSCLC	Non-small cell lung cancer
TNBC	Triple negative breast cancer
PARP	Poly(ADP-ribose) polymerase
Pd_2_(dba)_3_	Tris(dibenzylideneacetone)-dipalladium(0)
PI	Propidium iodide
Xphos	2-Dicyclohexylphosphino-2′,4′,6′-triisopropylbiphenyl
DCM	Dichloromethane
GAPDH	Glyceraldehyde 3-phosphate dehydrogenase
