# Supplementary material for: Novel and Potent Small Molecules against Melanoma Harboring BRAF Class I/II/III Mutants for Overcoming Drug Resistance
Source: Int J Mol Sci. 2021 Apr 6;22(7):3783. doi: 10.3390/ijms22073783 (PMC8038755; doi:10.3390/ijms22073783)
Supplement: Supplementary file 1 [file ijms-22-03783-s001.pdf]

Supplementary Materials

Table 1. Anti-proliferative activities of the six GNF-7 analogues on cancer cells harboring class II/II BRAF mutations and skin fibroblast cells.

Entry	GI_{50} (μM)[a]			
	Class II	Class II	Class III	-
	BRAF G464V	BRAF G469A	BRAF G466V	BRAF wt
	TNBC MDA-MB-231	NSCLC H1755	NSCLC H1666	skin fibroblast HFF-1
vemurafenib	13.65 ± 0.89	8.04 ± 0.83	> 50	19.76±3.06
PLX8394	17.86 ± 0.31	7.22 ± 1.28	36.65 ± 6.54*	>50**
GNF-7	0.13 ± 0.01**	0.04 ± 0.01***	0.11 ± 0.02****	0.72 ± 0.25**
SIJ1227	0.04 ± 0.01***	0.04 ± 0.01****	0.03 ± 0.00***	0.11 ± 0.00**
SIJ1281	0.03 ± 0.01***	0.04 ± 0.01****	0.07 ± 0.02**	0.16 ± 0.01***
SIJ1278	0.15 ± 0.00**	0.18 ± 0.01***	0.19 ± 0.05***	0.66 ± 0.05**
SIJ1777	0.05 ± 0.00***	0.01 ± 0.00***	0.05 ± 0.01****	0.19 ± 0.02**
SIJ1744	0.12 ± 0.00***	0.11 ± 0.01**	0.11 ± 0.02***	0.25 ± 0.05**
SIJ1748	0.08 ± 0.06***	0.06 ± 0.01**	0.15 ± 0.03***	0.17 ± 0.03**
SIJ1287	0.13 ± 0.01**	0.02 ± 0.01***	0.05 ± 0.01***	0.32 ± 0.05***

[a]GI50 represents the concentration which inhibits 50% of half-maximal growth. Each cells were treated with indicated compounds for 72 h. Average with standard deviation (n = 3, duplicate) are shown. Statistical significances were determined using a one-way ANOVA analysis (*$p < 0.05$, **$p < 0.01$, ***$p < 0.001$, ****$p < 0.0001$).

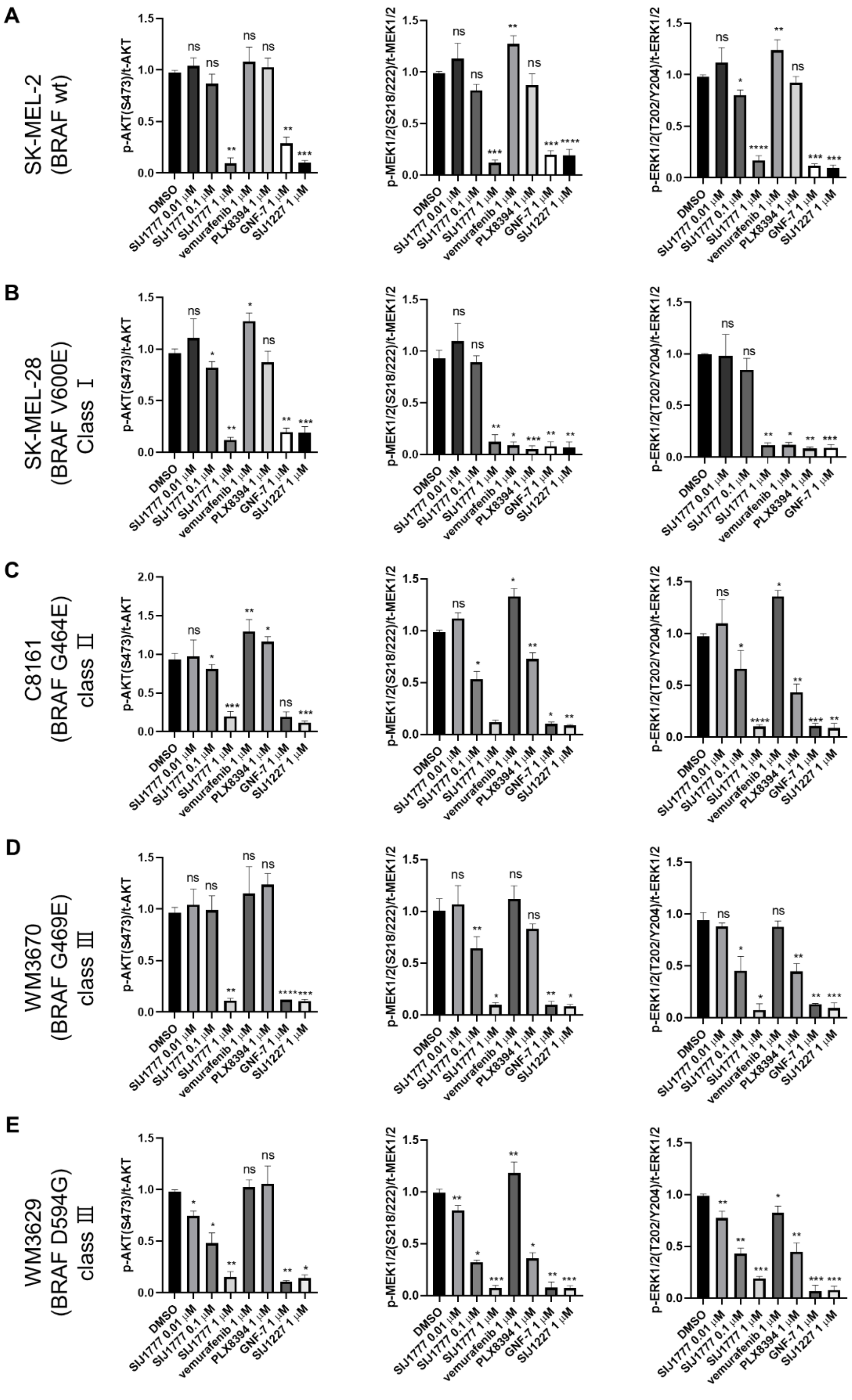

Figure S1. Quantitative analysis of western blot. Western blot images were automatically quantified by ImageJ (n = 3). Statistical significances were determined using a one-way ANOVA analysis (*$p < 0.05$, **$p < 0.01$, ***$p < 0.001$, ****$p < 0.0001$).

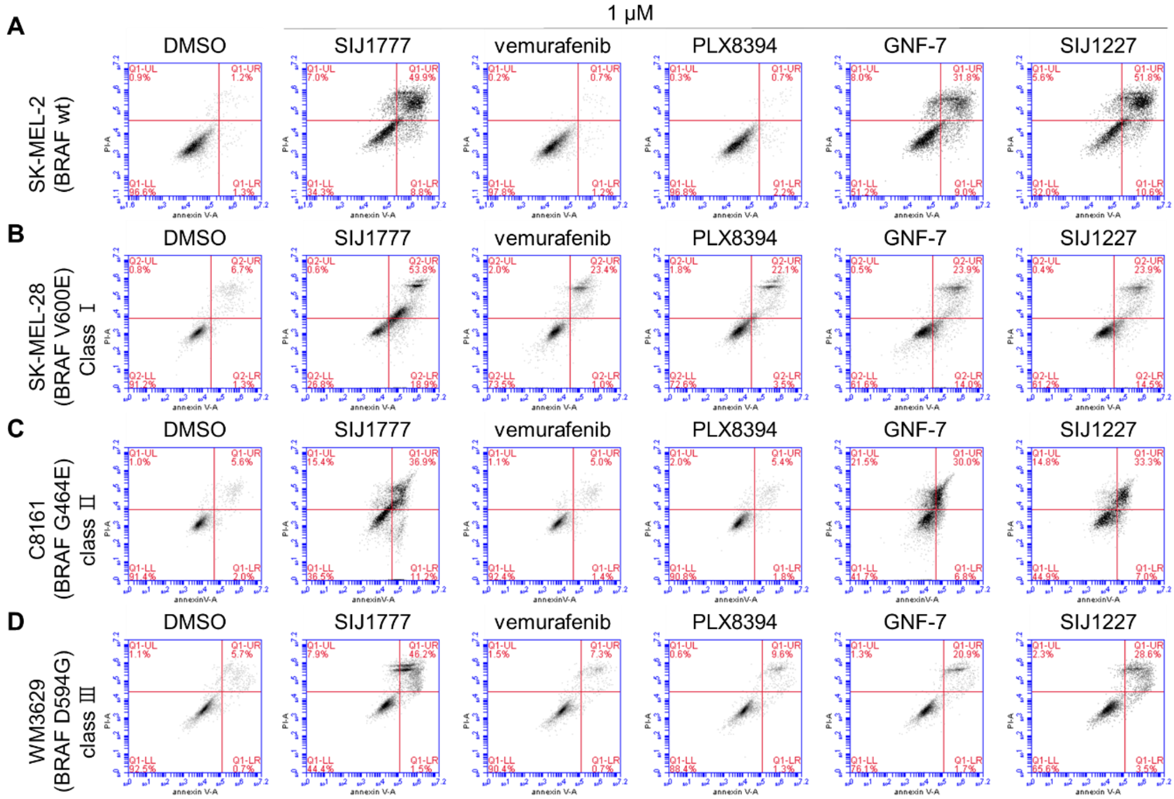

Figure S2. Apoptosis induction activity of SIJ1777 in melanoma cells. Cells were double-stained with Alexa Flour 488 conjugated annexin V and propidium iodide after compounds treatment for 24 h. FACS analysis were performed. (n = 3).

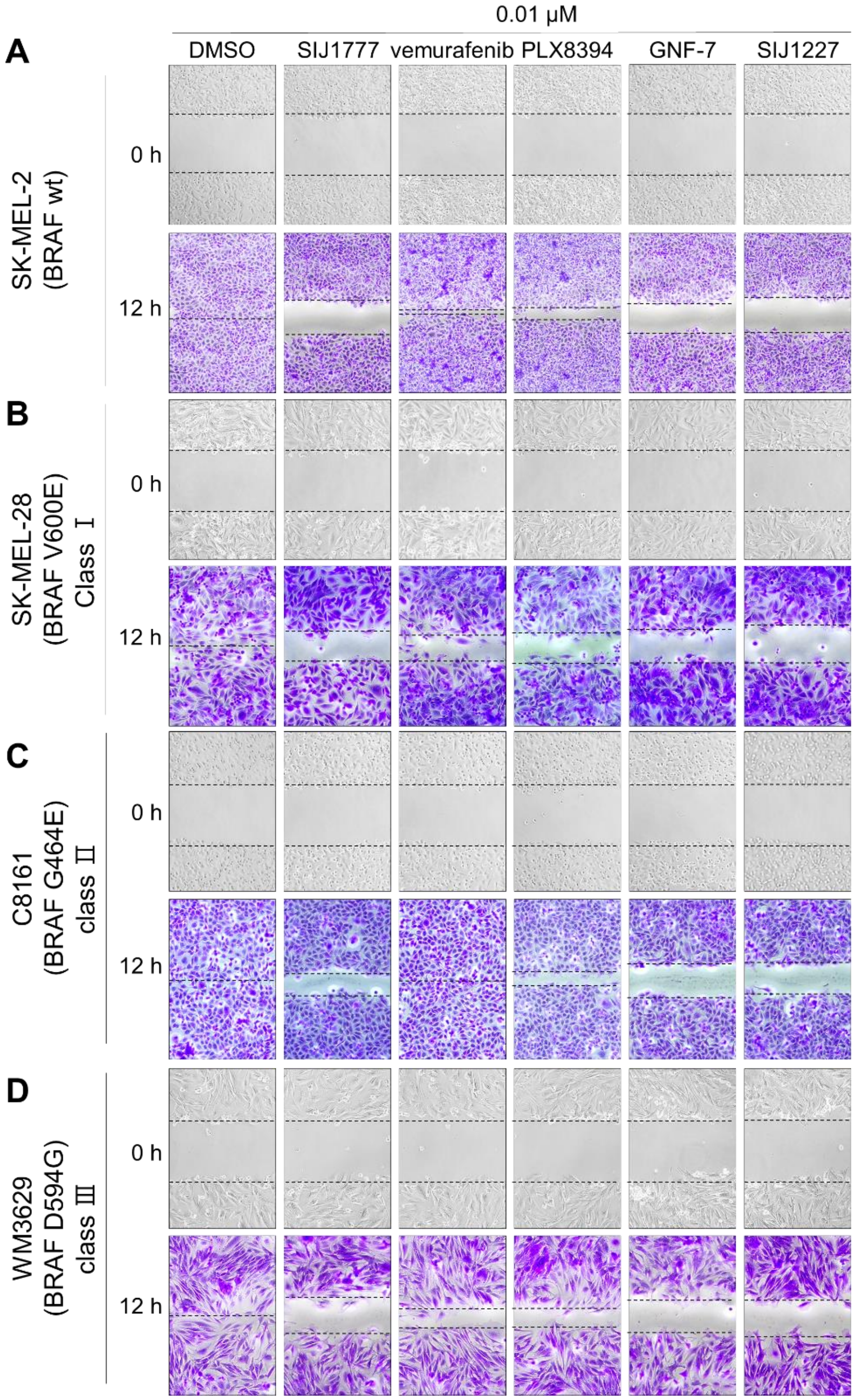

Figure S3. Migration inhibitory activity of SIJ1777 against melanoma cells. Scratch assay was performed for cell migration analysis. After scratching each cell monolayer, indicated compounds with 0.01 μM concentration were incubated for 12 h. Cells were photographed with 100 × magnification after staining with crystal violet solution for 20 min at room temperature.

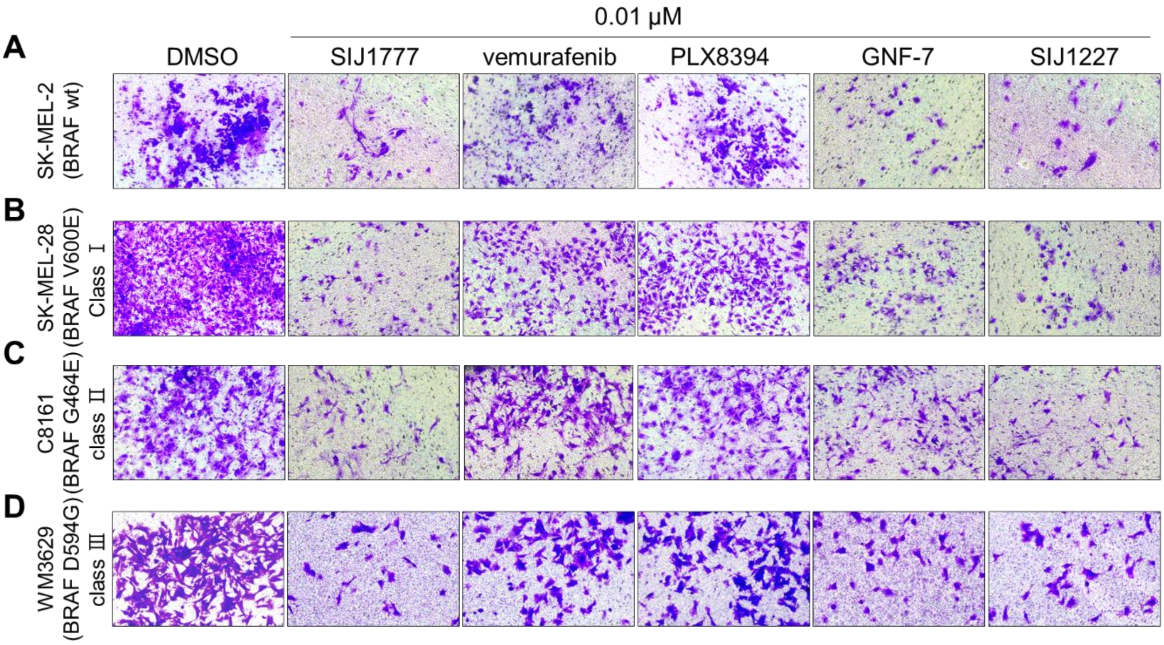

Figure S4. Invasion inhibitory activity of SIJ1777 against melanoma cells. Boyden chamber assay was performed for cell invasion analysis using cell invasion kit (QCM ECMatrix Cell Invasion Assay). Cells were photographed with 100 × magnification after staining with crystal violet solution for 20 min at room temperature.
